# Toxicity and *T*_2_-Weighted Magnetic Resonance Imaging Potentials of Holmium Oxide Nanoparticles

**DOI:** 10.3390/nano7080216

**Published:** 2017-08-07

**Authors:** Timur Sh. Atabaev, Yong Cheol Shin, Su-Jin Song, Dong-Wook Han, Nguyen Hoa Hong

**Affiliations:** 1 Department of Physics and Astronomy, Seoul National University, Seoul 08826, Korea; 2 Department of Cogno-Mechatronics Engineering, Pusan National University, Busan 46241, Korea; choel15@naver.com (Y.C.S.); songsj86@gmail.com (S.-J.S.)

**Keywords:** holmium oxide, paramagnetic nanoparticles, *T*_2_-weighted magnetic resonance imaging, toxicity

## Abstract

In recent years, paramagnetic nanoparticles (NPs) have been widely used for magnetic resonance imaging (MRI). This paper reports the fabrication and toxicity evaluation of polyethylene glycol (PEG)-functionalized holmium oxide (Ho_2_O_3_) NPs for potential *T*_2_-weighted MRI applications. Various characterization techniques were used to examine the morphology, structure and chemical properties of the prepared PEG–Ho_2_O_3_ NPs. MRI relaxivity measurements revealed that PEG–Ho_2_O_3_ NPs could generate a strong negative contrast in *T*_2_-weighted MRI. The pilot cytotoxicity experiments showed that the prepared PEG–Ho_2_O_3_ NPs are biocompatible at concentrations less than 16 μg/mL. Overall, the prepared PEG–Ho_2_O_3_ NPs have potential applications for *T*_2_-weighted MRI imaging.

## 1. Introduction

Magnetic and optical metal oxide nanoparticles (NPs) have attracted considerable attention over the past three decades for biomedical imaging and diagnosis [1]. In particular, iron oxide NPs [2,3], manganese oxide NPs [4,5], and gadolinium oxide NPs [6,7] have been investigated for potential magnetic resonance imaging (MRI). However, superparamagnetic iron oxide NPs show saturation magnetization at approximately 1.5 T, which limits their MRI applicability in high magnetic fields [2]. From this point of view, paramagnetic rare-earth metal oxide NPs with higher magnetic moments and higher density of magnetic ions per surface unit are more promising for MRI applications. For example, Gd_2_O_3_ NPs were reported to show higher longitudinal relaxivity (*r*_1_) compared to commercially available Gd-based chelates [8,9]. Gd_2_O_3_ NPs brightens the imaging place (positive contrast), because it changes the spin-lattice relaxation of water protons. On the other hand, the main limitation associated with the broad use of Gd_2_O_3_ NPs is in their high toxicity. In this regard, surface modification or Gd-doping into a less toxic material can be used [10,11], but these alterations can also deteriorate the relaxivity rates of the Gd_2_O_3_ NPs.

Other rare-earth ions, such as Dy^3+^ and Ho^3+^, have larger magnetic moments (~10.5 μ_B_) than Gd^3+^ (~8.1 μ_B_). On the other hand, both Dy_2_O_3_ and Ho_2_O_3_ NPs are more suitable for *T*_2_-weighted MRI (negative contrast) due to the fast spin relaxation of their 4*f* electrons. For example, a number of studies demonstrated the suitability of Dy_2_O_3_ NPs for *T*_2_-weighted MRI [12,13]. In particular, the reported transverse *r*_2_ relaxivities of Dy_2_O_3_ NPs were much higher than that of commercially available iron oxide NPs [12,13]. On the other hand, there are almost no reports of the potential toxicity and applications of Ho_2_O_3_ NPs as a MRI contrast nanoprobe [14]. Therefore, this study examined the PEG-grafted Ho_2_O_3_ NPs to explore their toxicity and applicability as a new potential *T*_2_-weighted MRI contrast agent. A murine fibroblast L-929 cell line was used as a pilot in-vitro model to check the cytotoxicity of the PEG–Ho_2_O_3_ NPs. This study suggests that the prepared PEG–Ho_2_O_3_ NPs can be potentially used as a new *T*_2_-weighted MRI contrast agent at concentrations less than 16 μg/mL.

## 2. Materials and Methods

### 2.1. Synthesis Process

Analytical grade Ho_2_O_3_ (99.9%), HNO_3_ (70%), polyethylene glycol (PEG, average M_n_ = 4000) and urea (99.0–100.5%) were purchased from Sigma-Aldrich (St. Louis, MO, USA) and used as received. Ho_2_O_3_ NPs were prepared using the reported protocols [15,16]. In brief, holmium oxide powder was converted to a holmium nitrate salt with the help of nitric acid. Later on, a sealed beaker with a freshly prepared aqueous solution of holmium nitrate (0.5 mmol in 40 mL of H_2_O) was placed into a forced convection drying oven (J-300M, Jisico Co., Ltd., Seoul, South Korea) and heated to 90 °C for 1.5 h. The collected precipitates were then calcined in air at 600 °C for 1 h to produce the Ho_2_O_3_ NPs. PEG-functionalization of Ho_2_O_3_ NPs was performed according to a reported protocol [8]. The obtained colloidal solution was then dialyzed in deionized ultrapure water for 24 h to eliminate the unreacted products.

### 2.2. Characterization

The structure of the prepared powders was examined by X-ray diffraction (XRD, Bruker D8 Discover, Billerica, MA, USA) using Cu-Kα radiation (λ = 0.15405 nm) at a 2θ scan range 20–60°. The morphology of the particles was characterized by transmission electron microscopy (TEM, JEM-2100, JEOL Ltd., Tokyo, Japan). Energy dispersive X-ray spectroscopy (EDX, JEOL Ltd., Tokyo, Japan) was used to perform an elemental analysis. Hydrodynamic sizes and zeta potentials of the obtained nanoprobes were measured using a Nano ZS Zetasizer (Malvern Instruments Ltd., Malvern, UK). Fourier transform infrared infrared spectroscopy (FTIR, Jasco FT/IR6300, Tokyo, Japan) was used to examine the structural properties of prepared samples. The magnetization measurements were performed using a magnetic properties measurement system (MPMS-5XL/Quantum Design Inc., San Diego, CA, USA). The *T*_2_-weighted images were obtained using a 1.5 T small animal MRI scanner (Siemens Healthinners, Enlargen, Germany). The measurement parameters used were as follows: the repetition time (TR) = 2009 ms, the time to echo (TE) = 9 ms, the field of view (FOV) = 160 × 160 mm, slice thickness = 5 mm, matrix = 256 × 256, number of excitations (NEX) = 1. All characterization measurements were performed at a room temperature of 22 ± 1 °C. The conditions for cell culture, cytotoxicity assay, fluorescence assay and statistical analysis were reported in our previous report [11].

## 3. Results and Discussion

**Figure 1:**
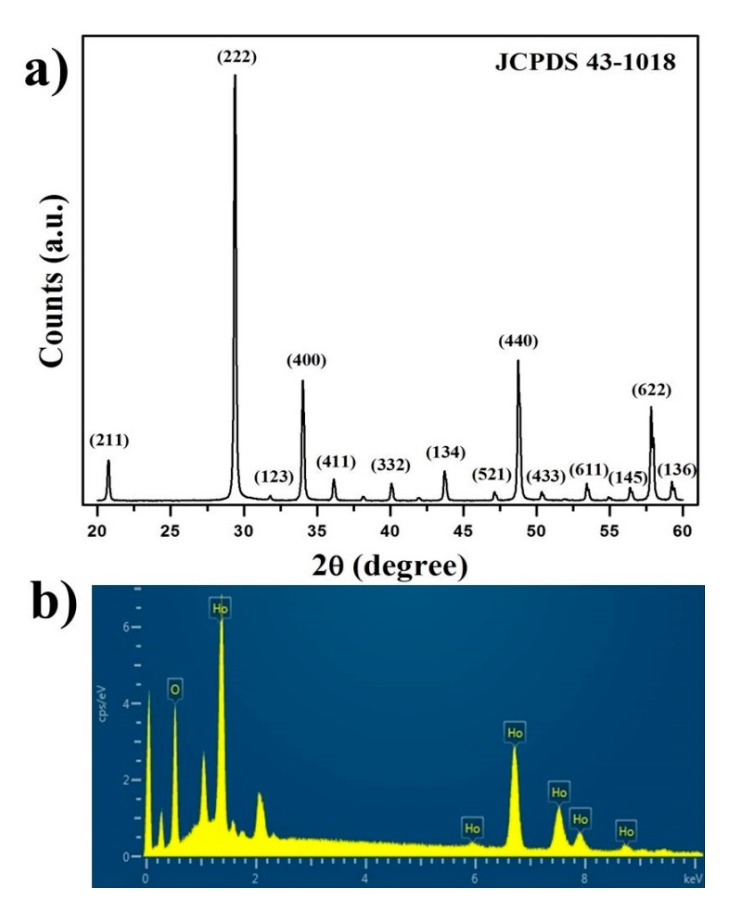
(**a**) X-ray diffraction (XRD) and (**b**) EDX analysis of as-prepared Ho_2_O_3_ nanoparticles (NPs).

Paramagnetic NPs for multimodal imaging have attracted considerable interest in recent years for potential nanomedical applications. Ultrasmall holmium oxide Ho_2_O_3_ NPs were proposed recently for potential MRI imaging applications [14]. However, the biocompatibility of Ho_2_O_3_ NPs is still a big issue to be addressed. Furthermore, it is very important to develop eco-friendly and low-cost synthesis method for fabricating the highly monodispersed Ho_2_O_3_ NPs at large scales. To address these issues, we designed a simple two-step approach to synthesize the highly monodispersed PEG–Ho_2_O_3_ NPs. The successful synthesis of PEG–Ho_2_O_3_ NPs is confirmed with several analysis techniques. XRD was used to examine the structural properties of the as-prepared Ho_2_O_3_ NPs. Figure 1a shows an XRD pattern of the as-prepared Ho_2_O_3_ NPs. The XRD peaks were assigned to the standard cubic (*Ia*_3_) Ho_2_O_3_ structure (JCPDS no. 43-1018) [17]. No additional impurity peaks were detected; thus, the obtained nanoprobes can be considered a pure cubic Ho_2_O_3_ phase. Energy dispersive X-ray spectroscopy (Figure 1b) revealed the presence of Ho and O elements only, indicating the formation of a pure Ho_2_O_3_ structure after a calcination process.

**Figure 2:**
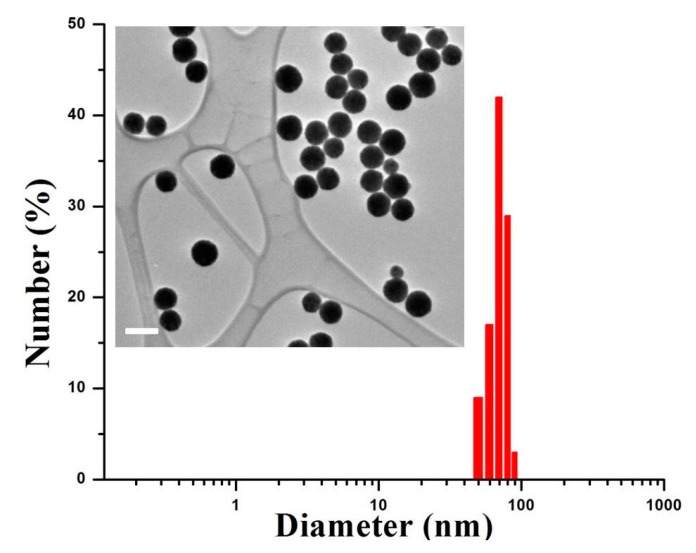
Measured hydrodynamic sizes of polyethylene glycol functionalized holmium oxide (PEG–Ho_2_O_3_) NPs. Inset is a transmission electron microscopy (TEM) image of PEG–Ho_2_O_3_ NPs (bar scale = 100 nm).

**Figure 3:**
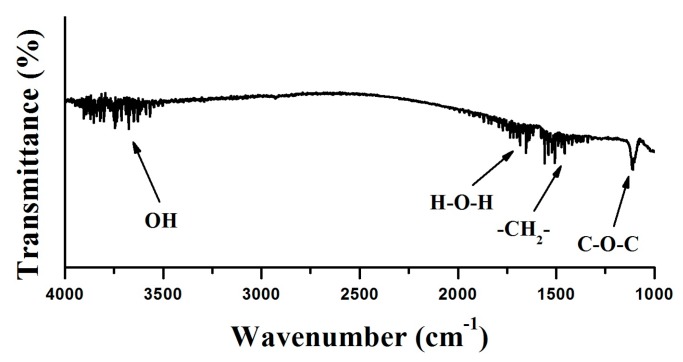
Fourier transform infrared spectroscopy (FTIR) analysis of PEG–Ho_2_O_3_ NPs.

Figure 2 presents the morphology and size distribution of the as-prepared PEG–Ho_2_O_3_ NPs. According to transmission electron microscopy, the prepared nanoprobes had an almost spherical morphology within the range 67–81 nm. On the other hand, the measured hydrodynamic sizes of PEG–Ho_2_O_3_ NPs were in the range of 80–90 nm (polydispersity index PDI = 1.67). The difference between observed and measured sizes can be explained by hydration coverage and existence of a thin PEG layer on the Ho_2_O_3_ NPs surface [18]. FTIR analysis (Figure 3) was used to examine the successful PEG-functionalization on the surface of Ho_2_O_3_ NPs. The PEG–Ho_2_O_3_ NPs showed the angular deformation of water molecules (~1660 cm^−1^) and the stretching vibrations of the OH group (~3600 cm^−1^). In addition, FTIR analysis showed the scissoring (~1470 cm^−1^) and waging (~1340 cm^−1^) modes of the CH_2_ group of the PEG chain. A most prominent peak at ~1100 cm^−1^ was also assigned to the PEG chain C–O–C vibration [8]. Thus, FTIR analysis confirmed the presence of water and PEG molecules on the surface of Ho_2_O_3_ NPs. A thin PEG layer on the Ho_2_O_3_ NPs surface can enhance the steric repulsion and prolong the blood circulation time [8]. In addition, one can achieve higher biocompatibility of prepared NPs through the PEG surface functionalization. The zeta potential was further measured at the physiological pH of 7.4 to ensure the colloidal stability of the PEG–Ho_2_O_3_ NPs. The measured zeta potential for PEG–Ho_2_O_3_ NPs was approximately (−16.7 mV). Therefore, the colloidal solution of PEG–Ho_2_O_3_ NPs can be stable for a relatively long time.

**Figure 4:**
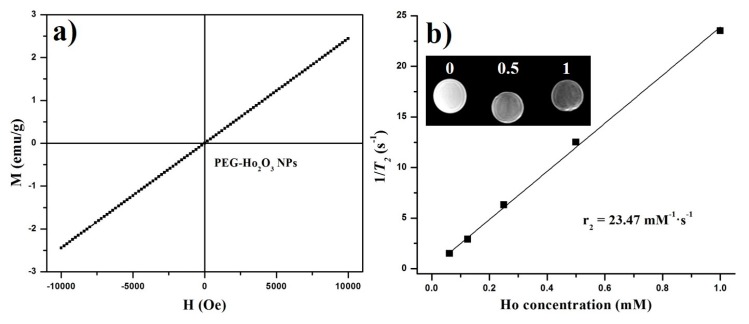
(**a**) Measured M(H) curve at 300 K; (**b**) r_2_ relaxation rate (1/*T*_2_) vs. Ho^3+^ concentration (mM). Inset is *r*_2_ map images of PEG–Ho_2_O_3_ NPs aqueous solution at different concentrations.

The magnetic properties of prepared PEG–Ho_2_O_3_ NPs were investigated further using an MPMS. Figure 4a shows the M(H) curve for the prepared PEG–Ho_2_O_3_ NPs at room temperature (*T* = 300 K). The observed linear relationship between the magnetization and applied field shows typical paramagnetic behavior of PEG–Ho_2_O_3_ NPs at room temperature. Figure 4b shows the measured inverse 1/*T*_2_ relaxation times vs. Ho^3+^ concentration. The transverse *r*_2_ relaxivity rate was estimated from a linear fit of 1/*T*_2_ vs. Ho^3+^ concentration. The slope of the linear fit revealed a transverse relaxation rate (*r*_2_) of 23.47 mM^−1^·s^−1^. One can also easily observe that the *r*_2_ map images become darker with increasing Ho^3+^ concentration (Figure 4b, Inset). The obtained *r*_2_ value of PEG–Ho_2_O_3_ NPs is much higher than the reported transverse relaxation rate (*r*_2_ = 17.95 mM^−1^·s^−1^) for Mn-doped iron oxide NPs [19]. It should be also noted that magnetic moment of Ho_2_O_3_ is not saturated at room temperature compared to widely employed iron oxide NPs [14]. As a result, the magnetic moment of PEG–Ho_2_O_3_ NPs will further increase with an increase in the applied magnetic fields. Therefore, the prepared PEG–Ho_2_O_3_ NPs can be applied as a *T*_2_-weighted MRI agent, particularly at high magnetic fields, because their contrast enhancements will increase with an increase magnetic field [12,13].

**Figure 5:**
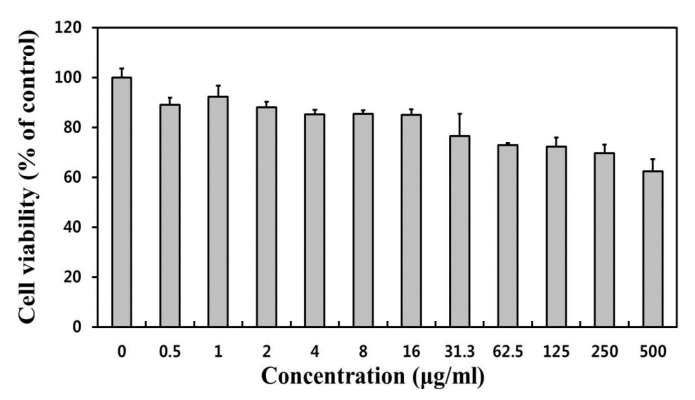
Cytotoxicity profiles of PEG–Ho_2_O_3_ NPs in L-929 fibroblastic cells.

The toxicity of the prepared NPs is another important factor that should be taken into consideration for potential nanomedical applications. Figure 5 presents the cytotoxicity profiles of the PEG–Ho_2_O_3_ NPs in L-929 fibroblastic cells using a WST-8 assay [10,11]. Metal ions can generate reactive oxygen species in the cell interior (“Trojan horse” mechanism), which leads to oxidative stress to living cells [20]. Therefore, the cytotoxicity results showed an obvious dose-dependent decrease in their relative cell viability. Obviously, PEG–Ho_2_O_3_ NPs caused no significant decrease in cell viability up to 16 μg/mL. Considering the in-vitro cytotoxicity only, the PEG–Ho_2_O_3_ NPs can be used at concentrations less than 16 μg/mL. However, the cytotoxicity against the cells exposed to PEG–Ho_2_O_3_ NPs must be tested by other viability end-point measurements.

**Figure 6:**
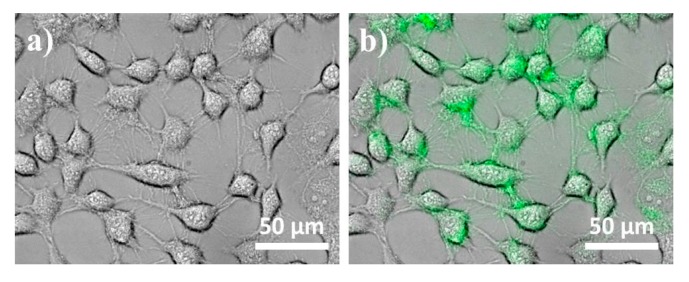
Phase contrast (**a**) and fluorescence (**b**) images of L-929 fibroblastic cells incubated with 10 μg/mL of PEG–Ho_2_O_3_ NPs.

Fluorescence microscopy (IX81-F72, Olympus Optical, Osaka, Japan) was used to visualize the cellular uptake and distribution of PEG–Ho_2_O_3_ NPs within the cultured L-929 cells. Figure 6a shows the phase contrast image of L-929 cells after incubation with PEG–Ho_2_O_3_ NPs suspension (10 μg/mL). The phase contrast image showed that the L-929 cells labeled with PEG–Ho_2_O_3_ NPs spread well with normal fibroblast-like morphologies. Although a detailed study for the cellular uptake was not performed, it is believed that the PEG–Ho_2_O_3_ NPs permeated into the cell membrane by non-specific endocytosis rather than pinocytosis [10,20]. Figure 6b shows that the prepared PEG–Ho_2_O_3_ NPs can also emit green light due to the intra 4*f*-transitions in holmium ions [21]. Therefore, prepared PEG–Ho_2_O_3_ NPs can be simultaneously utilized as a bimodal nanoprobe for MRI and optical imaging.

## 4. Conclusions

In summary, PEG–Ho_2_O_3_ NPs were prepared and their applicability as new *T*_2_-weighted MRI contrast nanoprobes was assessed. Cytotoxicity measurements showed that the prepared PEG–Ho_2_O_3_ NPs were nontoxic at concentrations less than 16 μg/mL. MRI relaxivity studies revealed high transverse relaxivity (*r*_2_ = 23.47 mM^−1^·s^−1^), suggesting that the prepared PEG–Ho_2_O_3_ NPs can be used as an efficient *T*_2_-weighted nanoprobe. In addition, green fluorescence was also detected from the PEG–Ho_2_O_3_ NPs due to intra 4*f*-transitions in holmium ions. Therefore, the prepared PEG–Ho_2_O_3_ NPs could be used as a dual-imaging nanoprobe.
